# Blood Biomarker Profiling and Monitoring for High-Performance Physiology and Nutrition: Current Perspectives, Limitations and Recommendations

**DOI:** 10.1007/s40279-019-01158-x

**Published:** 2019-11-06

**Authors:** Charles R. Pedlar, John Newell, Nathan A. Lewis

**Affiliations:** 1 Faculty of Sport, Health and Applied Science, St Mary’s University, Twickenham, UK; 2 Orreco, Business Innovation Unit, National University of Ireland, Galway, Ireland; 3 Division of Surgery and Interventional Science, University College London (UCL), London, UK; 4 Insight Centre for Data Analytics, National University of Ireland, Galway, Ireland; 5 School of Mathematics, Statistics and Applied Mathematics, National University of Ireland, Galway, Ireland; 6 English Institute of Sport, Bath, UK

## Abstract

Blood test data were traditionally confined to the clinic for diagnostic purposes, but are now becoming more routinely used in many professional and elite high-performance settings as a physiological profiling and monitoring tool. A wealth of information based on robust research evidence can be gleaned from blood tests, including: the identification of iron, vitamin or energy deficiency; the identification of oxidative stress and inflammation; and the status of red blood cell populations. Serial blood test data can be used to monitor athletes and make inferences about the efficacy of training interventions, nutritional strategies or indeed the capacity to tolerate training load. Via a profiling and monitoring approach, blood biomarker measurement combined with contextual data has the potential to help athletes avoid injury and illness via adjustments to diet, training load and recovery strategies. Since wide inter-individual variability exists in many biomarkers, clinical population-based reference data can be of limited value in athletes, and statistical methods for longitudinal data are required to identify meaningful changes within an athlete. Data quality is often compromised by poor pre-analytic controls in sport settings. The biotechnology industry is rapidly evolving, providing new technologies and methods, some of which may be well suited to athlete applications in the future. This review provides current perspectives, limitations and recommendations for sports science and sports medicine practitioners using blood profiling and monitoring for nutrition and performance purposes.

## Key Points

**Table:** 

Some blood biomarkers can be used for profiling and monitoring purposes in athletes, and the biomarkers selected depend on the demands of the sport.
Statistical methods for longitudinal data analysis are recommended to generate individualised thresholds to identify meaningful changes over time.
The insights gained from blood profiling and monitoring can provide an objective means of assessing nutritional status and capacity to tolerate training load.
Poor quality data will be generated if pre-analytic protocols are not carefully followed, for example, posture, time of day, recent food or exercise.

## Introduction

Many professional and Olympic-level athlete settings comprise comprehensive sports medicine and sports science support services, with an objective of: (1) achieving the highest possible level of performance with the lowest number of days lost to injury or illness [1], and (2) a duty of care to protect athletes from long-term negative health consequences of their sport [2]. A wealth of measurable variables of task-specific performance, training load, physiology, health and wellness exist to facilitate this, which can be used to guide coaches and athletes. In many cases this now includes blood profiling and monitoring, yet there has been no recent review of the practical application of blood profiling and monitoring in sport aimed at this interdisciplinary team. Here, we define ‘blood profiling’ as any blood testing where the data are applied beyond a medical diagnostic or anti-doping purpose. This includes the use of biomarkers to assess the efficacy of training interventions, inform nutritional strategies, and assess the capacity to tolerate training load. We define ‘blood monitoring’ as tests that are conducted frequently (e.g. once per micro-cycle) in order to describe the recovery status of the athlete.

There are a host of positive and negative outcome indicators that can be found within the blood that may corroborate or contrast with subjective athlete reports of performance readiness and symptoms, or other objective test data. These can help the practitioner decide whether an athlete is likely to be able to sustain or adapt to training/high performance or to assess the efficacy of an intervention. For example, a high testosterone-to-cortisol ratio suggests greater anabolic drive and has been strongly associated with positive training and performance outcomes [3]; chronically low energy availability (evident in a reduction in triiodothyronine as an example) reduces the ability to adapt to training [4], while also being a risk factor for bone stress injuries [5]; low iron status compromises the erythropoietic effects of altitude linked to endurance performance [6]; and vitamin D deficiency is known to compromise immunity, muscle repair and bone health [7, 8].

The aim of this review is to provide a useful practical guide to blood biomarker profiling and monitoring; it is not intended to be an exhaustive summary of the literature. It is beyond the scope of the present review to discuss sampling of other body fluids such as saliva, urine and tear fluid [9], or to discuss advanced techniques emerging in sports science such as metabolomics and ‘athleticogenomics’ [10–12]. However, this is not intended to diminish their future importance.

Importantly, there are a number of considerations that are often overlooked in the application of blood biomarker measurement in sport, including: (1) consideration given to what is ‘normal’ and what constitutes a meaningful deviation from normal for each individual athlete; (2) pre-testing considerations such as the time of day, posture, fasting/hydration status, transportation and storage of samples, the effects of recent training sessions (i.e. timeline for the restoration of homeostasis for each analyte); (3) sports-specific expertise present to interpret and address actions arising from testing; (4) appreciation of plasma volume shifts where the biomarker is volumetric in nature, e.g. haemoglobin.

### Screening Versus Monitoring

Depending on the frequency of measurement, essentially two approaches can be adopted. The first is screening, i.e. infrequent measurement of selected biomarkers (several months apart) to identify deficiencies or excesses; the second is monitoring, i.e. high-frequency measurement of biomarkers (days or weeks apart) in order to assess ongoing adaptation or recovery (readiness) from disturbed homeostasis. Once enough data have accumulated, sport- (and position-) and athlete-specific reference ranges can be applied. In order to optimise the timing and application of these two approaches, detailed knowledge of the athlete’s training and competition programme is required.

While each biomarker provides information about one or more physiological systems, the insights gained are narrow if only a single data point is available. Depending on the sport, sex, and the specific context, an appropriate biomarker or panel of biomarkers can be selected and measured at a suitable frequency. The success of a biomarker screening/monitoring programme depends on a number of factors, including the financial cost, validity and sensitivity (see Tables 1, 2).

**Table 1:** Key factors for the success of biomarker profiling in sport

Clinical oversight: collaboration between the sports doctor and the sports scientists
Selection of appropriate actionable biomarkers for screening and monitoring (see Table 2)
Appropriate frequency of testing
Sufficient financial resources to cover costs of collection, analysis, interpretation and feedback
Contextual information available to be used in interpretation
Implementing statistical best practice in data visualisation, modelling and translation
Availability of expertise to interpret biomarkers
Athlete and/or coach ‘buy-in’ and appropriate/effective feedback mechanisms

**Table 2:** Checklist of considerations for assessing biomarker suitability in sport

Evidence	Has prior research provided a satisfactory evidence base for the use of this biomarker (clinically, in public health or in sport), and for the specific target population and sex?
Application	Will the biomarker provide actionable data or serve as a useful positive or negative outcome indicator?
Validity	Has the biomarker been demonstrated to be valid? If this is a new technique, does it agree with established ‘gold standard’ technique?
Variability (analytical and biological)	Is the variability of this measurement technique acceptable (often reported as the coefficient of variation; CV). Has the analytical and biological variability of the biomarker been reported?
Collection and analysis	Is the collection procedure and analysis time fast enough to be useful? Is the amount of blood required appropriate? (i.e. minimal)
Sample treatment and transportation	Can the analysis take place *in situ*, or does the sample have to be stored in a specific way and/or transported to a laboratory
Diurnal variation	Does the time of day, exercise, sleep and fasting status influence the biomarker?
Cost	Is the full cost of the biomarker data justified?
Covariates	Are there factors that are known specifically to influence the biomarker? e.g. environmental impact such as warm weather camp, altitude, travel stress and jet lag

The usefulness of screening and monitoring with blood biomarkers in providing information that might ultimately reduce injury and illness risk, or impact upon the rate of adaptation to training, is a complex subject. The literature to date will not always provide a clear guide since large randomised, controlled studies of the behaviour of each biomarker are unlikely to ever be possible in these specialised populations. A needs analysis is a logical starting point for undertaking blood biomarker profiling. Over 3 decades of applicable studies of biomarkers in sport, together with extensive medical literature, exist for practitioners to draw upon to enhance decision making. In addition, biomarker technology is rapidly evolving, driven by the colossal biotechnology industry.

### Interdisciplinary Team Approach

The application of blood testing for sports performance often requires the complementary skillsets of the sports medicine doctor, sports scientists and biostatistician to work in collaboration. For the purpose of this review, the term ‘sport scientist’ might include associated disciplines of physiology, nutrition/dietetics, and strength and conditioning. The importance of these collaborations cannot be overstated because clinical oversight is required for all blood tests that might be diagnostic of pathology, and therefore due consideration must be given to medical liability. For example, if a clinical/pathological abnormality is uncovered during routine blood profiling, action is required by the sports medicine doctor to ensure optimal duty of care.

Statistical ‘best practice’ for the analysis of longitudinal data is needed in order to make informed decisions [13], with the contextual information provided by the sport scientist. Since athletes are often outliers, routine screening can create a high number of abnormal results for clinical diagnostic tests, albeit often of no clinical consequence (i.e. false positives [14]). Furthermore, on a practical level tests cannot typically be requested from a clinical laboratory without a medical doctor’s licence, although this varies considerably by location.

Athlete health is recognised as being closely linked to sustained high performance, and unfortunately some sports are known to be strongly associated with disease continuums either during or post career [15–17]. Reducing inflammation and oxidative stress (OS) [18] may be an important objective for protecting athletes from overt disease [19], or from sports-specific medical problems such as tendinopathy in basketball [20] or the deleterious effects of concussion [21]. Looking ahead, it seems appropriate for sports science, sports medicine and biostatistics to work closely together towards athlete health goals, and blood biomarker analysis provides a prime opportunity for such collaboration. Further studies are needed to demonstrate the effects of modifying biomarkers in competing athletes on career longevity and on post-career health.

### How Much Venous Blood is Reasonable to Remove from an Athlete?

It is widely accepted that small blood losses via phlebotomy are naturally replenished rapidly in the hours following a draw, at least among non-athletes. However, removing a significant quantity of blood on a regular basis could clearly be detrimental, and therefore minimising the amount of blood removed is advised. Red blood cells (RBCs) are released from the bone marrow at an estimated rate of > 2 million per second [22] to support a total blood volume of between approximately 4 l and 8 l, depending on body size and sport. Each cubic millilitre of blood contains 4–6 million RBCs, and over half of the sample is plasma, comprising > 90% water. Each 10 ml of venous blood drawn represents approximately 0.1–0.3% of total blood volume. To provide some context with regards to the impact of blood losses via phlebotomy, it is known that females are more susceptible to iron deficiency primarily due to menstrual blood loss, with loss estimated as light flow, < 36.5 ml; medium flow, 36.5–72.5 ml; and heavy flow, 72.5 ml per cycle [23]. A 26-night simulated altitude research study that clamped total haemoglobin mass (tHbmass) in a subgroup of endurance athletes, removed on average of 180 ml (range 82–314 ml) of blood via phlebotomy to negate hypoxia-induced erythropoiesis [24], resulting in a cancelling out of aerobic performance gains. This illustrates that the environment- or training-induced gains in tHbmass can be reversed with blood loss. Blood draw volume and frequency should therefore be kept to a minimum with a clear and well-justified purpose.

## Limitations of Blood Testing in Athletes

There are a number of practical limitations to blood testing, which are evolving as new technology emerges (see Sect. 3). Often the cost of testing can be prohibitive and therefore some kind of cost-benefit analysis is advised. The cost of tests varies vastly by country (e.g. clinical laboratory panels are considerably more expensive in the USA than in Europe) and by the specific test panels selected. The time between the blood draw and the arrival of results can vary considerably depending on the test and mode of measurement. Where delays occur, the analysis can only be retrospective, thus limiting the potential impact of the findings.

The tests themselves also carry limitations. For example, measuring haemoglobin concentration in a sample does not provide a measure of the tHbmass, since that is dependent upon blood volume and is affected by shifts in plasma volume [25] (see Sect. 8). Quantification of immune-cell populations is also limited since it does not provide data on the *function* of those cells, and cell populations have the propensity to migrate or translocate from the circulation [26]. Additionally, cells that reside outside of the circulation will not be detected with a blood test—for example, immune cells that reside in the skin [27].

For monitoring purposes, blood samples are routinely drawn with the athlete in a rested state. However, incorporating blood tests before and after controlled physical testing (e.g. a maximal aerobic capacity test or controlled training sessions) can provide additional insights from an athlete monitoring perspective. For example, the measurement of endocrine hormones after submaximal and maximal exercise is more effective in characterising fatigued states in endurance athletes than measures at rest [28]; hormonal responses to a two-bout exercise protocol can diagnose overtraining syndrome [29]; inflammatory cytokine responses to controlled treadmill running may differ between healthy and illness-prone athletes [30]; and the response in redox biomarkers to exercise is a well-established method used to assess OS [31] and more recently for predicting adaptation [32], with overloaded athletes displaying a diminished plasma antioxidant response to an exercise test [33]. Caution is warranted over applying an additional physical load purely for the purposes of monitoring, but carefully integrating specific monitoring variables around timed physical testing may be beneficial in managing athlete training load and recovery. An example of this may be conducting a routine training session in a controlled manner and measuring heart rate, rating of perceived exertion and blood biomarker responses.

## Evolving Biomarker Technology Available to Practitioners in Sport

Anecdotally, convenience is a major consideration in the success of biomarker measurement in athletes. Blood sample collection is now possible without traditional venepuncture via micro-filament needles inspired by mosquitoes [34, 35], although this technology has not yet been widely deployed. A continuum exists with comprehensive biomarker analysis via venous blood sampling at one extreme, and point-of-care tests for single biomarkers via capillary sampling at the other (lactate is the obvious example in sport, blood glucose is the most common point-of-care test globally). Additionally, some biomarkers can be assessed from a blood spot sample collected on filter paper—for example, red cell fatty acids. As the market for personalised medicine and the ‘quantified self’ has dramatically expanded with promise of a laboratory in one’s pocket [36], many companies have started offering extensive blood panels from small samples collected at home but often with compromised precision or accuracy. One such company, Theranos, was not only found to be less accurate than high-throughput laboratories [37], but was also recently exposed as fraudulent in the promise of comprehensive biomarker analysis from a finger-prick sample [38]. In this context, caution is warranted when selecting appropriate technology for use in sport. Table 2 provides a checklist for assessing the suitability of new blood-testing technology.

## Pre-analytic Considerations

The composition of blood is highly dynamic and never in a fixed state in vivo. Following collection, depending on the collection tube, blood cells continue to metabolise, the cells will begin to separate from the plasma, and the sample can coagulate. Therefore, the pre-analytic considerations are fundamental to achieving a suitable specimen and robust data. These are well-established phenomena [39], yet often overlooked in the sport setting.

**Fig. 1:**
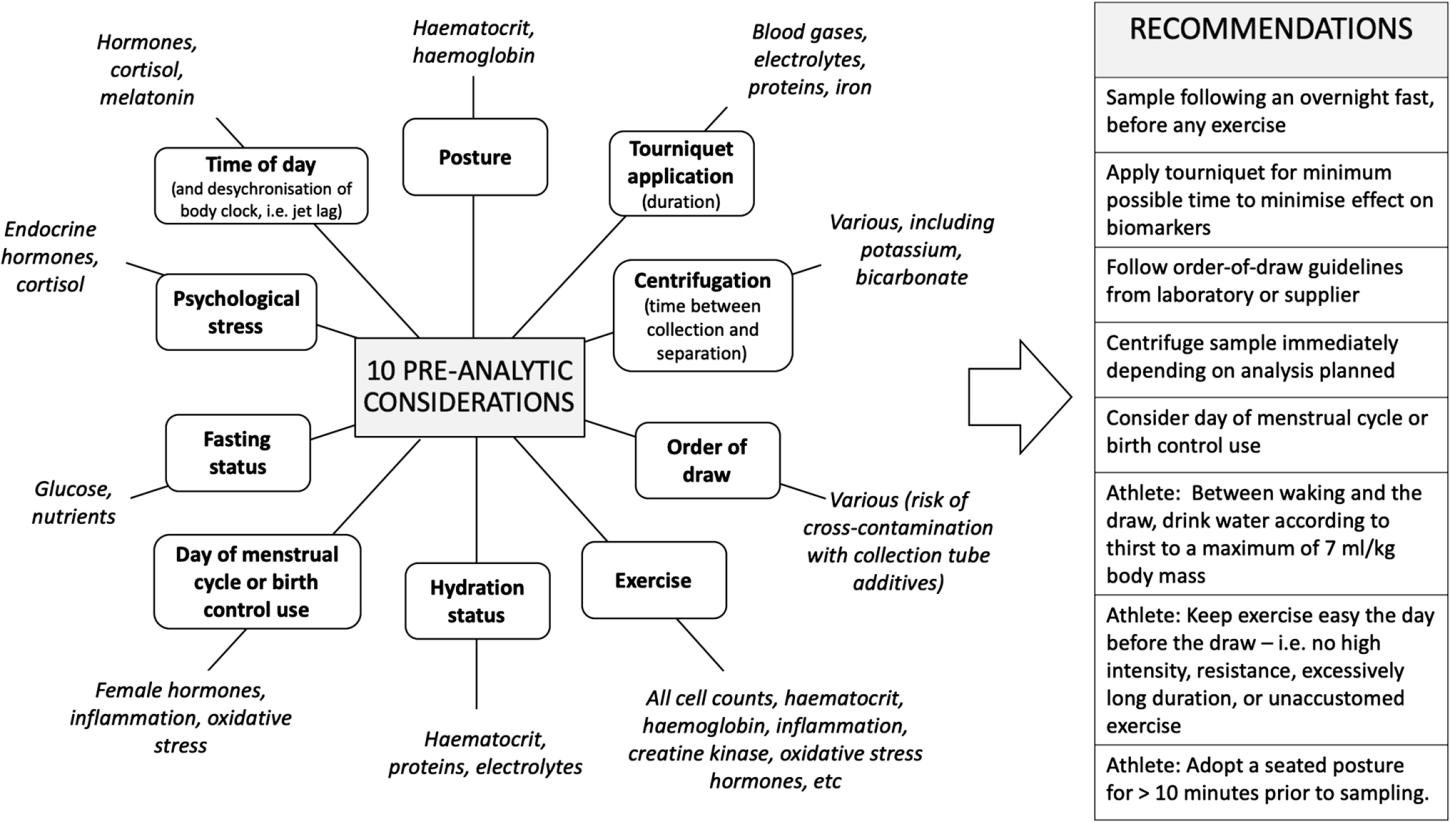
Pre-analytic considerations for the measurement of blood biomarkers from a venous blood sample. The recommendation regarding hydration is based on American College of Sports Medicine guidelines [139]

Here we define ‘pre-analytic’ as all factors that influence a blood specimen prior to analysis in the laboratory, displayed in Fig. 1. Posture (supine vs. seating vs. standing), duration of tourniquet application for venous samples, the separation of cells from plasma (i.e. the time of centrifugation), time of day, psychological stress, fasting status, day of the menstrual cycle, hydration status, and the duration, intensity and mode of prior exercise can all influence the data [40–42]. The relative impact depends on the test being conducted. Flouting these procedures in sport is tempting for convenience, but can result in dramatic inaccuracies in the data with ‘knock-on’ effects for subsequent data analysis.

## Statistical Considerations

Population-based medical reference ranges are typically generated using a cross-sectional sample from the general population and may not always be useful for interpreting athlete data. Furthermore, a ‘baseline’ value can be challenging to obtain in athletes with congested training and competition schedules and ubiquitous global training stress. In small samples with large between-subject variability, population-based reference ranges are often too wide to be informative. As examples, a recent study reported that male athletes with testosterone values in the lower quartile of the sample, but within the clinical range, had a 4.5-fold higher stress fracture rate [5]; hypervolemia associated with endurance training can dilute cell counts, giving a false impression of anaemia [43]. Published athlete data that could be used to create athlete reference ranges are generally absent, with some exceptions [44–48]. A sport or governing body regularly collecting data on a specialised group of athletes might rapidly accumulate a suitable dataset in-house, as published by the Australian Institute of Sport some 2 decades ago [48].

**Fig. 2:**
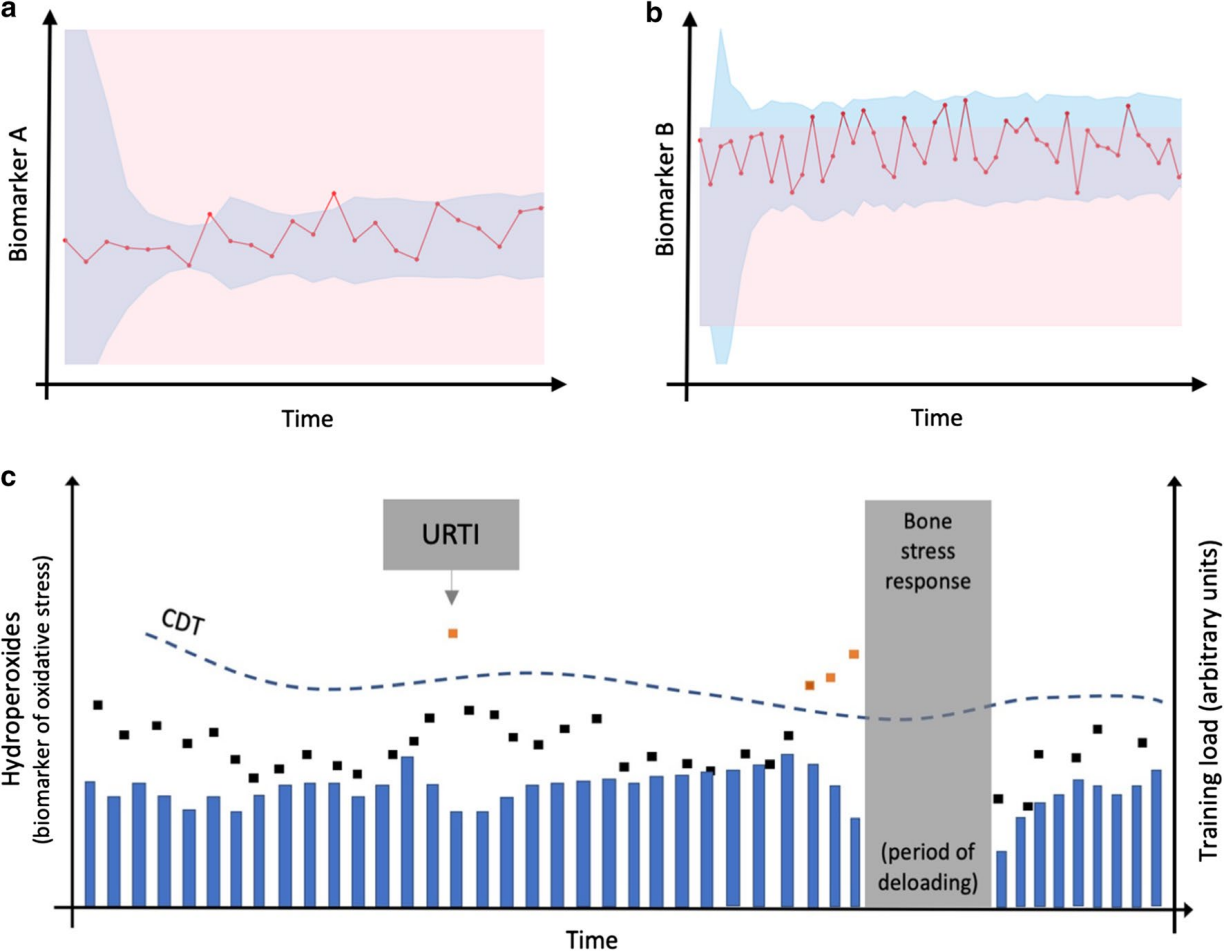
Charts (**a**) and (**b**) illustrate biomarkers collected repeatedly over time (red lines). The rectangular shaded areas represent a population based clinical range for this biomarker; the blue shaded areas represent an individual Bayesian adaptive range. Chart (**c**) illustrates a biomarker of oxidative stress (hydroperoxides; black and orange squares) collected frequently with blue bars representing a global marker of training load for each microcycle. *URTI* upper respiratory tract infection, *CDT* critical difference threshold

Monitoring, by its nature, requires statistical methods for longitudinal data analysis. For example, a Bayesian approach considers prior information (i.e. knowledge about the biomarker distribution), to categorise new data and identify data points of interest. The reference range generated adapts dynamically as more information on the athlete’s within-subject variability is available. This is the approach employed to create the adaptive individualised ranges used in the athlete biological passport [49]. These individualised approaches are used to identify atypical measures by providing adaptive rather than static reference ranges, and are of higher potential value to the sports science team [50–52]. Examples of the application of individualised ranges are provided in Fig. 2a, b.

A calculated critical difference threshold (CDT) may be useful in monitoring situations whereby the known variance due to biological variation and measurement error is quantified and applied to create an individual CDT for each analyte [50]. With the CDT, a greater degree of confidence can be achieved in understanding whether a ‘true’ physiological change has occurred for the analyte in question [50, 53]; see Fig. 2c. Ideally, the CDT should be calculated in the athletic group of interest to minimise physiological differences as a source of error. Other methodological approaches (e.g. index of individuality) are available for assisting practitioners in evaluating the usefulness of population-based biomarker reference intervals for interpreting change in individuals [50].

Modelling biomarkers jointly (and not marginally) over time using suitable multivariate statistical techniques in combination with training, wellness and other data sources has received little attention in sports science to date, but could be of value in the future for the purposes of objectively managing training load, identifying injury and illness risk, and predicting performance.

## Specific Examples of Blood Testing for Nutrition Purposes

### Using Blood Profiling to Inform Nutritional Recommendations

The dietary habits of athletes are assessed in order to construct individualised dietary plans designed to optimise training responses, performance and health. There are limitations associated with the various commonly applied qualitative methodologies (i.e. dietary recall, food frequency questionnaires, diet diaries) [54]. For example, in an individual male, in order to estimate his true average intake of iron with a degree of confidence, 68 days (range 13–130 days) of food intake records would be required (see Basiotis et al. [55]). Blood profiling, however, provides an efficient, reliable, quantitative means of assessing nutritional status (both deficiencies and excesses), which is not subject to reporting bias.

Nutritional blood biomarker profiling may be used to assess compliance and a response to a given dietary intervention (e.g. serum carotenoids following an increase in fruit and vegetable consumption), and to ascertain whether timely nutritional adjustments are required to optimise recovery and adaptation (e.g. thyroid hormones with reference to energy availability during a period of intense training; see Sect. 7). Although many nutrients are well researched in sport, there are some exceptions—for example, iodine, which is well known to have an interaction with exercise and to be lost via sweat [56].

Many nutritional markers are not well suited to blood profiling since their concentration in the blood is small in comparison to specific tissue compartments—for example, serum calcium, which does not reflect calcium status [57], and serum magnesium (Mg), for which the gold standard is a 24-h urine collection following an oral Mg loading dose [58]. Conversely, other nutrient blood tests such as measurement of fatty acids incorporated in RBC membranes [59], glycated haemoglobin (HbA1c) and red cell Mg reflect dietary exposure over the life of the RBC and therefore provide useful indices of global dietary habits.

Since the measurement of biomarkers relating to nutrition is described in detail elsewhere [54], we instead will address other, more novel nutritional biomarkers that have not been described in detail elsewhere in the sports medicine literature, including RBC fatty acids, biomarkers of fruit and vegetable intake, and biomarkers of amino acids.

### Red Blood Cell Fatty Acids

Consumption of dietary fats can be assessed through the analysis of RBC fatty acids via a dried blood spot technique [60], although it should be acknowledged that endurance training alters skeletal muscle membrane phospholipid composition through an increase in docosahexaenoic acid (DHA) content [61]. Skeletal muscle phospholipid eicosapentaenoic acid (EPA) and DHA are strongly correlated to RBC phospholipid EPA and DHA (*r *= 0.913) [62]. RBC fatty acids are responsive to changes in the intake of fish, olive oil and fish oil supplements [63, 64]. The omega-3 index (OM3I), a validated, reliable and reproducible biomarker for the assessment of omega-3 status, represents the percentage of the long chain marine fatty acids EPA and DHA as a proportion (%) of the total RBC fatty acids [59]. Data are now available in athletic populations: a mean (standard deviation) of 5.1 (1.0)% in Summer Olympians [65], 4.9 (1.2)% in Winter Olympians [66] and 4.4 (0.8)% in National Collegiate Athletic Association Division 1 collegiate footballers [67]; however, wide inter-athlete variability was consistently observed. These findings in athletes contrast with an average OM3I of 3.7 (1.0)% in a large cohort of vegans, 3.5 (0.7)% in US military servicemembers, and a median OM3I of 7.1% in a Spanish cohort consuming a Mediterranean diet [68–70]. Currently, the recommended target range for OM3I in athletes is 8–11% [66]. However, there is no experimental evidence to date in athletes to substantiate such a precise claim for health or performance; further research in this area is warranted.

Healthy college students with an OM3I above 4% experienced significantly lower post-eccentric exercise muscle soreness (DOMS) at 72 and 96 h, lower 24-h C-reactive protein concentrations, and improved profile of mood states compared to the ‘low’ OM3I group (< 4%) [71]. Increasing the OM3I from ~ 4.5 to ~ 6% in endurance athletes through supplementation enhanced cycling economy [72], and in a military study, a relationship was observed between OM3I (within a narrow OM3I range of 2–5%) and cognitive flexibility and executive function [70]. Together, these studies suggest that measuring and manipulating OM3I in athletes may be a useful endeavour to augment both health and performance, although further studies in well trained and elite athletes are needed to clearly establish cause and effect, particularly given the capacity for training to alter skeletal muscle phospholipid composition [61].

### Biomarkers of Fruit and Vegetable Intake

Fruits and vegetables (FV) contain an array of polyphenols, vitamins, minerals and fiber, and are essential to athlete health, recovery and performance. The measurement of serum carotenoids constitutes a valid means for the assessment of FV intake [73]. Studies deploying a short-term (2-week) restriction of FV intake (i.e. a low antioxidant diet: restricted to one serving of fruit and two servings of vegetables per day) in athletes resulted in substantial decreases in resting serum carotenoid concentrations, along with increased exercise-associated lipid peroxidation with exercise, increased ratings of perceived exertion (RPE), and increased resting and exercise inflammatory responses [74, 75]. A comparable low antioxidant diet in asthmatics resulted in a decline in serum carotenoids and decreased lung function [76]. Moreover, increasing athlete phytonutrient (FV, nuts and seeds) intake has been observed to substantially increase serum carotenoid concentrations and contribute to enhanced recovery and performance in a world-class endurance athlete [53]. Specific training paradigms such as ‘live-high, train-low’ may lead to decreases in serum antioxidant vitamins and carotenoids [77, 78]. It follows that modifying these variables may support athlete recovery and health, although further studies are needed. These studies relate to dietary fruit and vegetable intake, and for clarity it should be noted that this is not synonymous with high-dose antioxidant supplementation where there is a well-established risk of blunting adaptation [79].

OS is affected by a broad range of factors, such as diet, lifestyle, environment and training, and OS biomarkers (of which there are many, and beyond the scope of this review) have been extensively researched in athletes (see Lewis et al. [80] and Finaud et al. [81]). OS biomarkers are modifiable through diet [74, 75], and vitamin insufficiencies (e.g. vitamin C) increase OS and decrease physical performance [82]. Recent studies have recognised the importance of identifying a blood redox profile for an individual (i.e. the existence of a low, medium or high level of oxidative stress, and/or antioxidant enzyme or nutrient) in order to identify those individuals in whom physical performance may be enhanced through the correction of the redox ‘deficiency’ with the appropriate treatment, i.e. antioxidant [32, 83]. The administration of *N*-acetylcysteine (NAC) to a group with ‘low’ red blood cell glutathione (GSH; a ubiquitous antioxidant enzyme) improved both aerobic and anaerobic capacity, whereas an adverse effect was observed for NAC on aerobic performance in the ‘high’ GSH group [83]. Similarly, vitamin C supplementation improved physical performance in those with low but not high plasma vitamin C concentrations [82]. Measuring biomarkers of redox status may therefore aid in the individualisation and frugal use of antioxidant supplementation.

### Biomarkers of Amino Acids

Exercise training is known to alter plasma blood amino acid concentrations, with chronically fatigued elite athletes reported to have significantly different resting concentrations to some healthy elite athletes [84]. Over the past 25 years, two amino acid biomarkers in particular—glutamine (GLN) and glutamate (GLU)—have been researched as a method of monitoring for fatigued states in athletes, with noteworthy observations [84–89].

Briefly, prior to the 1992 Barcelona Olympics, both acutely fatigued and chronically fatigued elite athletes were screened and observed to have significantly lower plasma GLN than healthy non-fatigued elite athletes (a diet low in protein may have been a contributing factor [84]). The ratio of GLU to GLN consistently showed promise for monitoring training stress. Indeed, a number of authors in different locations [87–89] demonstrated significant changes in the plasma GLU/GLN ratio in national and international athletes, well-trained endurance cyclists, and team sport athletes during periods of intensified training.

Unfortunately, from a practical standpoint, assays of any amino acid are not readily available in clinical or commercial laboratories, which may explain the lack of recent research. Additionally, recent advances in approaches to periodising protein intake [90] around training load may serve to reduce the need for GLU/GLN monitoring. Metabolomic studies are emerging and may reinvigorate this field [91], although metabolomic data so far are currently sparse in sport.

## Assessing Energy Availability

Assessing energy availability is desirable to avoid the risk of the female athlete triad or the broader relative energy deficiency in sport (RED-S) theoretical framework [17, 92]. We have previously documented the importance of measuring bioenergetic hormones in athletes in order to protect the athlete from the deleterious effects of unexplained underperformance syndrome (also known as overtraining syndrome), of which chronic low energy availability (LEA) is a major risk factor [93]. LEA was strongly associated with athlete illness in the lead-up to a summer Olympic Games [94] and was associated with a 4.5-fold higher risk of bone injuries in both male and female distance runners with LEA [5]. There are a number of ways to estimate energy availability, such as monitoring changes in body mass, or by calculating energy availability as the difference between total energy intake and estimated energy output; however, the latter can be a time- and resource-consuming endeavour and there are a number of sources of potential inaccuracies associated with both these methods. Screening for energy availability indirectly with blood profiling is therefore a recommended approach [95].

Endocrine biomarkers, including the male and female sex hormones and thyroid hormones free triiodothyronine (free T3) and total triiodothyronine (TT3), offer insight into energy availability [96]. Although the benefits of using hormonal biomarkers as part of an athlete wellness/nutritional screening process are becoming more evident, tracking intra-individual changes through various training and competition phases may provide more meaningful data (enabling a shift from the dependence on clinical ranges for interpretation; see Sect. 5), and thus enabling physicians, sports practitioners and coaches to make timely adjustments to training and nutritional programs in order to optimise recovery and adaptation.

In addition, it is recognised that experienced elite male and female athletes do not self-adjust their energy intake during periods of intensified training, the outcome of which is a deterioration in performance [97]. A training study in female swimmers elegantly demonstrated the clear dependence upon sufficient energy availability for training success by monitoring a group of swimmers across a 12-week training block [4]. Five athletes with normal ovarian hormone cycles (estradiol and progesterone) were compared with five athletes with suppressed ovarian hormones and a significantly lower energy availability. Furthermore, 400-m swimming performance (velocity) improved in the energy-replete swimmers but not the energy-deficient swimmers despite completing the same training distance. Both bioenergetic hormones (TT3 and insulin-like growth factor-1) showed a significant decline in the energy-deficient swimmers only. While the absence of fluctuation in ovarian hormones is a useful marker of energy status in itself, the impact of the oral contraceptive pill can mask sex steroid differences, resulting in an advantage for measuring the bioenergetic hormones.

Although published data are undeniably limited in male athletes, poor energy availability and hormonal suppression (hypogonadism) may occur with persistently excessive endurance exercise and/or inadequate energy intake, and thus there is a parallel with the female athlete triad [98]. Significant changes over time in bioenergetic (free T3) and stress (cortisol) hormones during intensified training have been reported in male rowers, albeit performance was not assessed [99]. Hypogonadism has been documented in male Ironman athletes attending the World Championships [100] and in a case study of an elite mixed martial arts athlete [101]. Such case studies provide for ‘real-world’ insight. Kasper et al. [101] succinctly captured the severe negative effects of making weight and the gross energy deficiency on endocrine function (testosterone, cortisol, IGF-1) across 8 weeks; both health and performance were negatively affected in conjunction with the hormonal disturbances. Furthermore, military studies (in males) tracking bioenergetic and steroid hormones over periods of basic training clearly demonstrate the significant effects of a combination of stresses (intensified training, sleep loss and energy deficiency) on these hormonal systems [102]. Finally, carbohydrate restriction can significantly affect testosterone and cortisol responses to intense training in male athletes [103].

Physiologically relevant changes in IGF-1, thyroid hormones, testosterone and cortisol are observed in short time frames (e.g. 1 week), with marked recovery when nutrition and energy status are restored, demonstrating the sensitivity of these hormones to nutritional interventions.

## Oxygen-Carrying Capacity and Red Blood Cells

Haemoglobin is the oxygen-carrying protein in the RBC, containing iron-rich heme sub-units. A higher total tHbmass enables a greater maximal oxygen-carrying capacity and therefore a higher aerobic power. Endurance athletes have been reported to have around a 40% higher tHbmass than the general population [104], and many invest considerably in altitude training, aiming to further increase their tHbmass. Unfortunately, haemoglobin concentration in a blood sample is poorly correlated with tHbmass since this is dependent upon blood volume and is susceptible to dilution from plasma volume expansion with heat acclimation or prolonged exercise [104–106]. Carbon monoxide rebreathing has become the method of choice for measuring tHbmass in research settings and some sports institute settings; however, it requires specialist equipment and technical skills [25]. A recent attempt has been made to estimate plasma volume based on a host of biochemical markers, and the results are promising [107]. Sixty-eight percent and 69% of the variation in plasma volume was explained by eight and 15 routinely measured biomarkers, respectively, e.g. salts. It remains to be seen if this approach will be verified by further studies, but the potential is enticing, since tHbmass could be estimated from plasma volume estimates and haematocrit measurements. This opens the possibility of estimating aerobic capacity from a single blood test, which would be ground-breaking in both athlete monitoring and anti-doping.

Compromised iron status can affect both male and female athletes [45, 108] and can result in a sub-optimal tHbmass, with a recent study neatly demonstrating the effects of correcting an iron deficiency via supplementation [109] when using tHbmass as the outcome measure. In severe iron deficiency (ferritin < 12 ng mL^−1^), dramatic increases in tHbmass were demonstrated via supplementation [109]. Using blood-profiling data alone, the response to supplementation is more difficult to quantify. RBC data including the mean corpuscular volume and the mean corpuscular haemoglobin provide an indication of compromised erythropoiesis due to iron deficiency [110]. Similar variables in the reticulocytes (depending on the analyser used [110]) can also provide evidence of compromised iron status. Measurement of the peptide hormone hepcidin, although not yet widely available, shows promise as a highly informative addition to an iron panel in athletes, since it can define an individual’s propensity to absorb iron and has an interaction with exercise, iron deficiency and iron overload [111, 112]. For a comprehensive review of the identification of iron-deficient states, see Archer and Brugnara [113]. In athletes, altitude training represents a risk factor for iron deficiency, and following a blood test iron supplementation should be considered in this context where appropriate [6]. Other factors in athletes such as footstrike haemolysis, excessive sweating and dietary factors may also compromise iron status [108].

## Using Biomarkers to Assess Training Capacity and Manage Workload

Fine margins exist between the training dose necessary for adaptation and that which elicits maladaptation at the elite level, paralleling the theory of hormesis [114, 115], where a moderate dose of a stressor combined with effective recovery results in an adaptive response, but an excessive dose is maladaptive (synonymous with ‘overcooking it’). There has been a great deal of attention on the acute : chronic workload as a predictor of injury, with recent thinking recognising that covariates such as stress, sleep and age are potentially of equivalent importance [116]. Although more research is needed, blood profiling and in particular blood monitoring, in conjunction with workload and wellness data, can offer an objective tool for identifying capacity to train and recover in the context of a multiplicity of stressors, and can therefore be used to enhance the management of athlete workload schedules.

The timely point-of-care measurement of capillary blood biomarkers of muscle damage (e.g. creatine kinase), OS (biomarkers of pro-oxidant and antioxidant activity), inflammation (e.g. C-reactive protein, pro-inflammatory cytokines) and anabolic or catabolic status (e.g. cortisol, testosterone, urea) can provide data that may help sport scientists to assess individual tolerance of training and therefore propensity for successful adaptation, and inform the recovery needs of the athlete.

It is well known that intense exercise causes transient exercise-induced muscle damage (EIMD), and this is proportional to the stress imposed, particularly eccentric muscle loading [117–119]. A transient increase in creatine kinase can be expected with EIMD, which returns to baseline within 60 h depending on the physical insult and training status. Inflammation may also occur with EIMD to varying degrees, and there are many studies to support this [120, 121]. Athletes therefore can be expected to routinely have higher concentrations of creatine kinase [44], and this may be more pronounced during intense or unaccustomed training, for example during pre-season training.

Physiological stress, i.e. a disturbance in homeostasis, is a desired outcome of training in order to trigger adaptation. OS has been termed a ‘molecular switch’ [122] for upregulating antioxidant systems for healthy adaptation and avoidance of disease [114, 115]. However, where an imbalance occurs between stress and recovery, negative outcomes can ensue, such as maladaptation (performance plateau) [123] and fatigue, as several overload studies have demonstrated in endurance athletes [124, 125].

Other activities can cause augmented stress or reduce the rate of recovery—for example, long-haul travel where biomarkers with a strong circadian effect can be influenced, for example testosterone and cortisol and the so-called ‘sleep hormone’ melatonin [126]. Sleep quantity (and quality), a primary variable that influences recovery, can also impact upon a biomarker profile. Sleep loss is associated with elevated cortisol [127] and inflammation markers that are reversed with extra recovery sleep [128].

The team sport athlete (e.g. soccer player) is subject to various forms of stress (physical, psychological, lifestyle) over the course of a season that vary according to the professional league, player experience, position, fitness and individual adaptability. The daily monitoring of elite players’ workloads through objective (e.g. global positioning systems) and subjective measures (e.g. daily readiness to train responses) is pervasive in elite soccer [129], with biomarkers predominately used for health- and nutrition-screening purposes. However, the weekly application of biomarker monitoring has gained increasing traction at the elite level in team sports.

Several studies have explored the effect of a single soccer match on the recovery time course of markers of muscle damage, inflammation and OS, in which elevations may persist for 24–74 h post-match depending on the biomarker, recovery time between matches (micro-cycle), playing standard, sex and position [119, 130–133]. Others have recorded significant OS biomarker changes in relation to measures of workload (i.e. muscle damage; internal load) across various time points of the season in elite soccer players [134, 135]. In addition, biomarker investigations over a season in other team sports, such as professional rugby [136] and handball [137], corroborate observations in professional soccer that periods of OS occur in association with periods of higher training loads and competition.

## Conclusions and Future Directions

There are early signs of new ‘-omics’ science in sport [91, 138], but these are a long way from becoming the norm. Similarly, new technology that analyses an athlete’s blood without the need for traditional venepuncture is in existence and could eventually become commonplace in sport.

Blood biomarker science in elite and professional sports is rapidly evolving and can provide objective data for an interdisciplinary sports science and medicine team to support athlete health, nutrition and performance across a broad spectrum of physiological systems. Some nutritional biomarkers are well established (e.g. vitamin D and iron), whereas others need further research (e.g. fatty acids) to demonstrate their utility in sport. A range of biomarkers can provide information relating to athlete readiness to train, including biomarkers of OS, inflammation, protein turnover and hormones. New methods to estimate plasma volume using groups of biochemical markers show promise and may provide a new method for monitoring changes in an athlete’s aerobic fitness.

The success of a blood-biomarker profiling or monitoring programme in sport is dependent not only on the selection of appropriate biomarkers, but also upon the timing of the testing, successful interdisciplinary collaboration, appropriate longitudinal statistical methods and pre-analytic protocols.
